# Editorial: Adenine nucleotides in immunity and inflammation

**DOI:** 10.3389/fimmu.2025.1585484

**Published:** 2025-03-12

**Authors:** Christa E. Müller, Santina Bruzzone, Andreas H. Guse

**Affiliations:** ^1^ Pharmaceutical Institute, Pharmaceutical & Medicinal Chemistry, University of Bonn, Bonn, Germany; ^2^ Department of Experimental Medicine – DIMES, University of Genova, Genova, Italy; ^3^ The Calcium Signaling Group, Department of Biochemistry and Molecular Cell Biology, University Medical Center Hamburg-Eppendorf, Hamburg, Germany

**Keywords:** adenine nucleotides, immunity, inflammation, intracellular signaling, extracellular signaling, purine P2X receptors, ectonucleotidases

Extracellular and intracellular adenine nucleotides (ANs) act as paracrine mediators or intracellular second messengers in almost all central processes in biology and medicine. They are essential for many (intra)cellular communication processes and are ubiquitously found in the plant and animal kingdoms. Many aspects of AN-governed or -modulated cell-cell communication and intracellular signaling are only partially known. Moreover, the physiological and pathological roles of ANs are only beginning to be understood. The Research Topic ‘*Adenine Nucleotides in Immunity and Inflammation’* comprises reviews discussing the current state of the field, in addition to original research articles that clarify specific aspects of AN functions, or describe novel methods or tools for studying ANs in health and disease.

## Intracellular signaling

Intracellular signaling encompasses two broad areas, Ca^2+^ signaling evoked or modulated by ANs, and signaling by the intracellular second messenger 3’,5’-cyclic adenosine monophosphate (cAMP).

Ca^2+^ signaling often starts locally and, through several amplification mechanisms, subsequently turns into global signaling. Ca^2+^ microdomains are local Ca^2+^ signals that can be observed using high- or super-resolution microscopy imaging techniques. A review by Gil Montoya et al. described the use of mathematical modeling as a ‘lens’ to overcome the resolution limitations of modern microscopy by predicting AN-driven signaling processes. Along these lines, an open-source Python pipeline for offline Ca^2+^ microdomain analysis was presented by Woelk et al. as a tool to serve the community. A specific example of how ANs differentially regulate even the same cation channel was discovered by Pick et al.; the authors described the kinetics and current amplitudes of the non-selective cation channel TRPM2 over a wide range of Ca^2+^ concentrations, showing more rapid kinetics and higher amplitudes for the TRPM2 super agonist 2’-deoxy-ADPR as compared to ADPR.

In addition to Ca^2+^ signaling, which is known to be essential for T cell activation, cAMP is currently considered to be more of a negative intracellular regulator of T cell activation. The role of cAMP-metabolizing phosphodiesterases (cAMP-PDE) was reviewed by Bielenberg et al. A specific example of an orphan G-protein-coupled receptor (GPCR) linked to cAMP generation in T cells was presented by Krieg et al.

## Extracellular signaling

### Purine P2X receptors

Three papers are focused on the ATP-activated pro-inflammatory P2X receptor subtypes P2X4 and/or P2X7, and their role on immune cells.

Longo et al. discovered a critical role of the P2X7 receptor for antigen-specific T cell immunity in a viral infection model. Sierra-Marquez et al. studied the localization of P2X4 and P2X7 receptors in native mouse lungs; they found no evidence for heteromeric P2X4/P2X7 receptors or direct interaction of both receptor subtypes. In a study by Brock et al., the role of P2X4 and P2X7 receptors during CD8^+^ T cell activation was investigated using time-resolved high-resolution imaging. The study demonstrated that P2X4 and P2X7 receptor signaling enhances initial Ca^2+^ events during CD8^+^ T cell activation and plays a crucial role in regulating downstream responses, including NFAT-1 translocation, cytokine expression, and proliferation.

### Ectonucleotidases

Extracellular ANs are substrates of ectonucleotidases, nucleotide-metabolizing enzymes that are integrated into the cell membrane or attached to it. The enzymes can also be present in exosomes or as soluble enzymes in the extracellular fluid. They hydrolyze ANs by cleaving phosphoric acid ester groups. Depending on the subtype, ectonucleotidases convert ANs to other ANs, or form, at the end of the hydrolytic cascade, immunosuppressive, anti-inflammatory adenosine.

In their review article, Winzer et al. summarized the role of extracellular vesicles containing ectonucleotidases and other purine-metabolizing enzymes involved in purinergic signaling.

An original article by Zubiar et al. focused on the ectonucleotidase CD38 (NAD-glycohydrolase, also displaying ADP-ribosyl cyclase and cyclic ADP-ribose hydrolase enzymatic activities; CD, cluster of differentiation). CD38 deficiency in a mouse model of chronic graft-versus-host disease resulted in an altered transcriptomic response to the induction of the disease. These results indicate an immunomodulatory and pro-inflammatory role of CD38 in the onset of this disease model.

Jaeckstein et al. studied the key ectonucleotidase CD73 (ecto-5’-nucleotidase), which catalyzes the hydrolysis of AMP to produce adenosine. Utilizing knockout mice deficient in vascular endothelial CD73 expression, the authors investigated the role of the enzyme in the context of adipose tissue homeostasis.

### Adenosine receptors

Extracellular ATP, ADP, AMP, or NAD can be hydrolyzed by the orchestrated action of ectonucleotidases to ultimately produce adenosine, which triggers the activation of adenosine receptors (classified into four subtypes, A1, A2A, A2B, and A3). Extracellular adenosine regulates a wide range of physiological processes, such as the modulation of the immune system, with adenosine being mostly known as an immunosuppressive agent.

Wendlandt et al. demonstrated that the A_2A_ adenosine receptors are present in astrocytes in the olfactory bulb, one of the brain regions that is first affected in neurodegenerative diseases. Activation of A_2A_ adenosine receptors determines an increase in the intracellular cAMP concentration in both control and a mouse model of multiple sclerosis, indicating that A_2A_ receptor-dependent pathways in olfactory bulb astrocytes are not affected by neuroinflammation.

Paladines et al. demonstrated that the anti-inflammatory action exerted by extracellular adenosine in human gingival fibroblasts is mediated by the activation of A_2A_ receptors and by subsequent triggering of the AMP kinase/Sirtuin 1/PGC-1α axis, with upregulation of mitochondrial biogenesis, ultimately regulating mitochondrial oxidative phosphorylation and metabolic energy production.

## Methods, technologies and tools

Several studies describe new methods and tools to study AN signaling and their targets in inflammation and immunity.

Hiefner et al. developed a liquid chromatography-tandem mass spectrometry-based method for the quantification of ANs and their degradation products. Stamataki et al. presented a review on novel adeno-associated viral vectors for the targeting of microglia: developing more precise, specific and effective interventions to modulate microglial behavior may represent a promising strategy to halt neuroinflammation. Finally, Lopez et al. studied natural and synthetic heparins as potent inhibitors of the AN-metabolizing enzyme ectonucleotide pyrophosphatase/phosphodiesterase-1 (NPP1). The authors concluded that this mechanism of action may contribute to the anti-cancer effects of heparins observed in some studies.

## Conclusion

In addition to their being crucial in metabolic energy, ANs are pivotal mediators of both extracellular and intracellular signaling. Both the release of ATP or NAD and their conversion by ectoenzymes to AMP/adenosine activate purinergic receptor signaling. These and other signaling pathways are coupled to the intracellular generation of AN second messengers that regulate Ca^2+^ homeostasis and cell responses. The exact understanding and definition of the molecular cascade governing physiological and pathological processes (inflammation, neurodegeneration etc.), along with the characterization of the expression of AN-degrading enzymes and AN-sensitive receptors in different conditions, may offer the possibility to develop a therapeutic strategy and/or to identify new biomarkers (possibly also expressed on EV). This Research Topic presents interesting contributions that cover different aspects of this multifaceted AN-based signaling network.

